# Mammary myofibroblastic sarcoma with lung metastasis: a case report and literature review

**DOI:** 10.3389/fsurg.2025.1698795

**Published:** 2026-02-12

**Authors:** Miri Ryu, Youn Joo Jung, Seungju Lee, Seok Kyung Kang, Meehyun Lee, Jee Yeon Kim, Kyung Jin Nam, Kyeyoung Lee, Ji Hyeon Joo, Jae Joon Kim, Hyun Yul Kim

**Affiliations:** 1 Department of Surgery, Research Institute for Convergence of Biomedical Science and Technology, Pusan National University Yangsan Hospital, Pusan National University School of Medicine, Gyeongnam, Republic of Korea; 2 Department of Pathology, Pusan National University Yangsan Hospital, Pusan National University School of Medicine, Gyeongnam, Republic of Korea; 3 Department of Radiology, Pusan National University Yangsan Hospital, Pusan National University School of Medicine, Gyeongnam, Republic of Korea; 4 Department of Radiation Oncology, Pusan National University Yangsan Hospital, Pusan National University School of Medicine, Gyeongnam, Republic of Korea; 5 Department of Hematology and Oncology, Pusan National University Yangsan Hospital, Pusan National University School of Medicine, Gyeongnam, Republic of Korea

**Keywords:** breast, lung, metastasis, myofibroblastic, sarcoma

## Abstract

Myofibroblastic sarcomas of the breast are rare, leading to very few reports on their prognosis and metastasis potential. We report a case of lung metastasis from a mammary low-grade myofibroblastic sarcoma. A 55-year-old woman with no remarkable past medical history presented to a local clinic with a palpable mass in her left breast that had persisted for 3 weeks. Vacuum-assisted excisional biopsy initially suggested a diagnosis of a malignant phyllodes tumor. Subsequent wide excision with clear surgical margins led to a confirmed diagnosis of myofibroblastic sarcoma. No additional treatments, such as chemotherapy or radiotherapy, were administered. Four years later, lung metastases were detected on chest computed tomography. The patient subsequently underwent wedge resection of the lung. Three years later, metastasis to the cervical lymph nodes was discovered. Owing to tumor emboli, the patient ultimately died from brain infarction. No consensus guidelines for standard treatment exist due to the scarcity of the disease. Although surgical resection remains the most reasonable treatment option, additional therapies should be considered to reduce the risk of metastatic spread to other organs.

## Introduction

Myofibroblastic sarcoma is an uncommon tumor that is rarely found in the breast (1), more typically arising in the extremities and the head and neck region, including the oral cavity and tongue (2). Low-grade myofibroblastic sarcoma (LGMFS) is characterized by myofibroblastic differentiation, locally infiltrative growth, and generally low metastatic potential; however, its clinical behavior in the breast remains poorly understood due to its rarity (3). To date, only a limited number of LGMFS cases have been reported in the literature (1, 4–6).

Owing to the scarcity of reported cases and absence of standardized treatment guidelines, previously described patients have undergone heterogenous management strategies, including variations in surgical extent and the selective use of adjuvant therapies. Long-term outcomes have likewise been variable, with some patients developing delayed metastases to distant organs. In this context, we present a case of mammary LGMFS with lung and cervical lymph node metastasis and provide the longest follow-up reported to date, highlighting the need for a better understanding of its metastatic behavior and clinical course.

## Case presentation

A 55-year-old woman presented to a local clinic with a palpable painless mass in her left breast that had persisted for 3 weeks. She first noticed the mass incidentally during self-examination. The patient had no significant past medical history, history of trauma or prior radiation exposure, and no family history of breast cancer or sarcoma-related malignancies.

Physical examination revealed a palpable mass without tenderness in the subareolar region of the left breast. Ultrasonography revealed a heterogeneous mass measuring 2 cm in diameter in the left subareolar area, and core needle biopsy (CNB) suggested hemangiopericytoma. The patient then underwent vacuum-assisted excisional biopsy, which revealed a malignant phyllodes tumor. She subsequently visited our center for further evaluation.

Preoperative laboratory test results were unremarkable. Breast mammography (MG, 3Dimensions) revealed a 1.9-cm, irregular, indistinct isodense mass with internal macro- and microcalcification and nipple retraction in the left subareolar region; the breast composition was categorized as type C. No abnormally enlarged lymph nodes were found in either axilla. Contrast-enhanced magnetic resonance imaging (MRI) revealed a symmetric, 1-cm, irregular, rim-enhancing mass with minimal background enhancement in the left subareolar region. Preoperative chest computed tomography (CT) and positron emission tomography (PET) showed no evidence of metastasis.

The patient underwent wide excision of the left breast with sentinel lymph node biopsy. Frozen-section analysis was also performed on the excision margins and two sentinel lymph nodes. All magnifications were clear, with no tumors.

**Figure 1:**
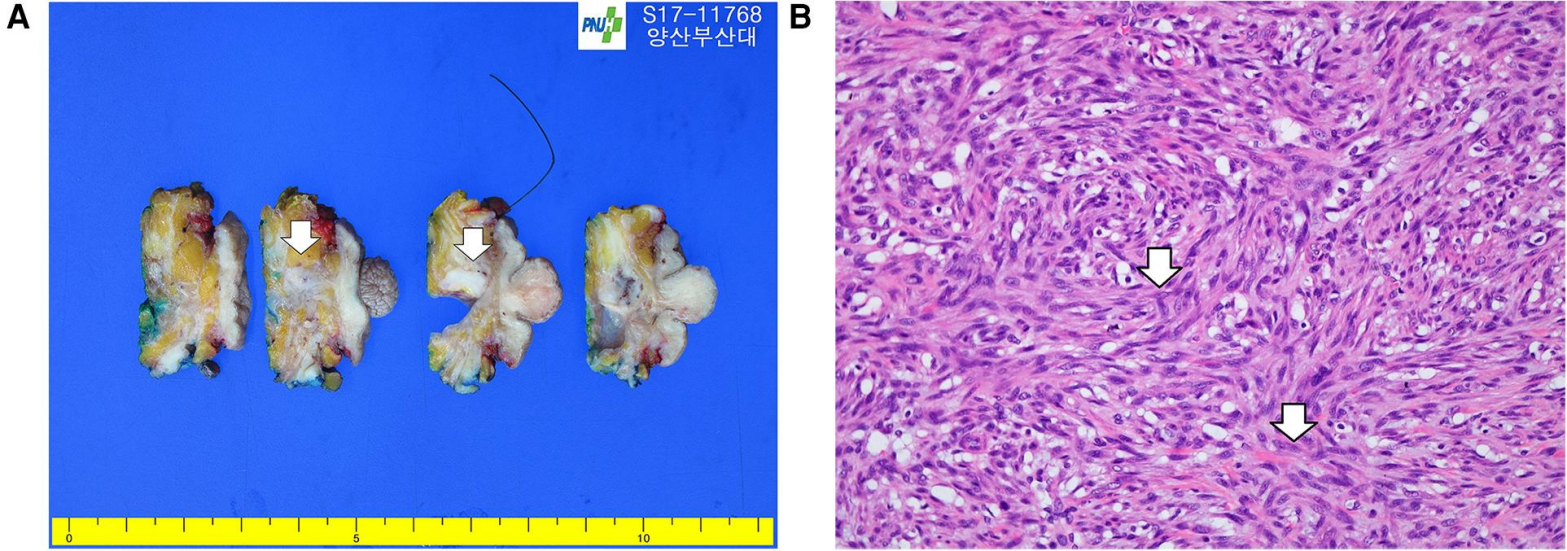
**(A)** Primary low-grade myofibroblastic sarcoma of the breast: The excised surface of the breast shows a relatively well-defined grayish-tan firm mass (white arrows). **(B)** Higher magnification showing storiform growth of spindle tumor cells (white arrow), characterized by ill-defined eosinophilic cytoplasm and a mitotic figure (white arrow) (40×, hematoxylin and eosin).

Permanent biopsy confirmed a diagnosis of LGMFS. The tumor measured 2.0 cm × 1.0 cm × 0.8 cm, and the cut surface of the breast specimen showed a relatively well-defined, grayish-tan, firm mass (Figure 1A). Higher magnification showed fascicles of atypical spindled tumor cells having elongated nuclei with irregular nuclear membranes. The tumor was composed predominantly of spindle-shaped cells. Due to the prominent storiform pattern, tumor cells were sectioned in both transverse and longitudinal sections; those transected transversely appeared round, whereas those sectioned longitudinally showed an elongated, cigar-shaped morphology (Figure 1B). Lymphovascular invasion was not identified (negative sentinel lymph nodes, 0 of 2). The closest resection margin measured 2 mm.

**Figure 2:**
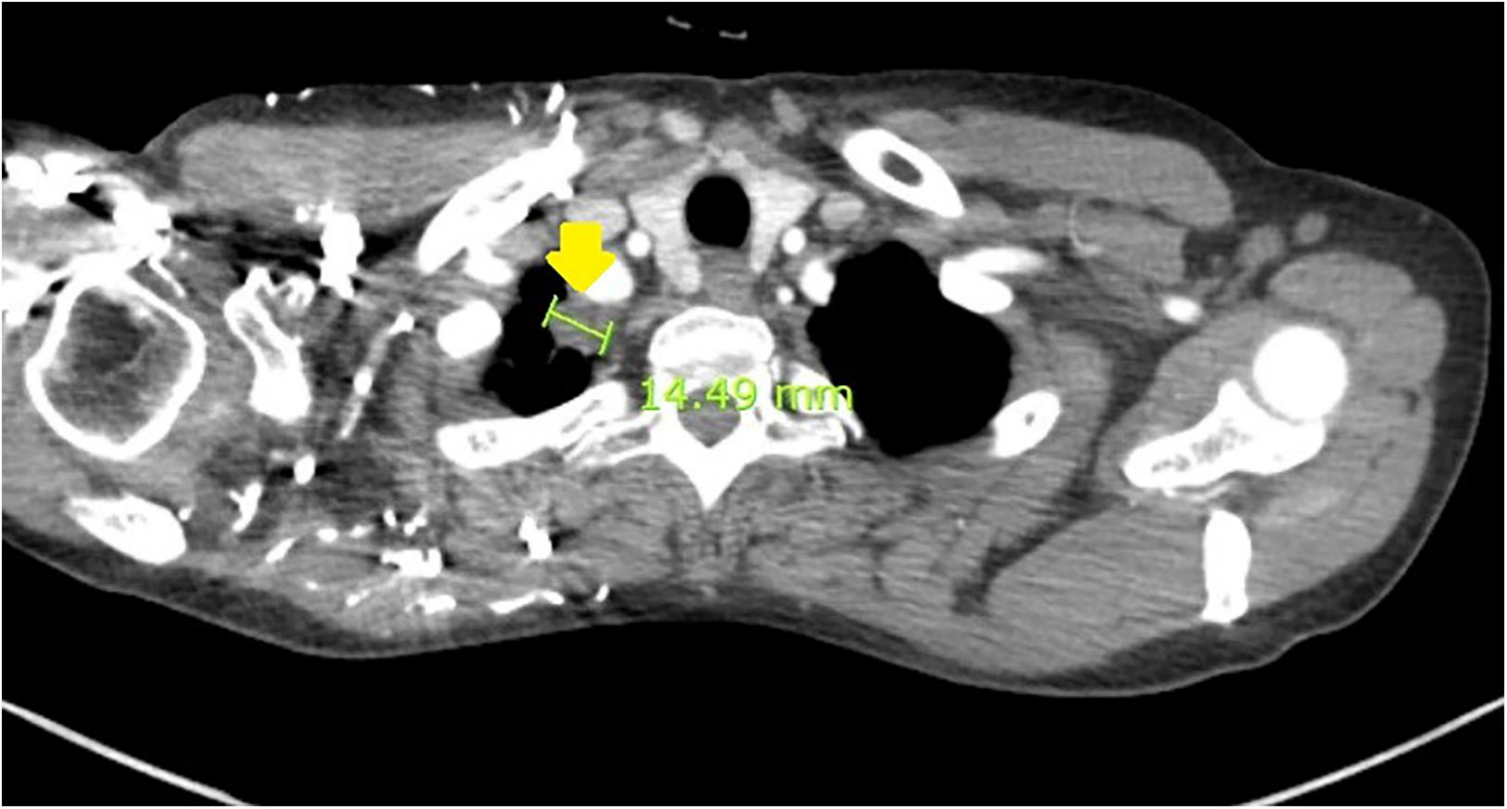
Chest CT showing an apical 1.5-cm mass (yellow arrow) and enlarged lymph nodes in the right upper lung.

**Figure 3:**
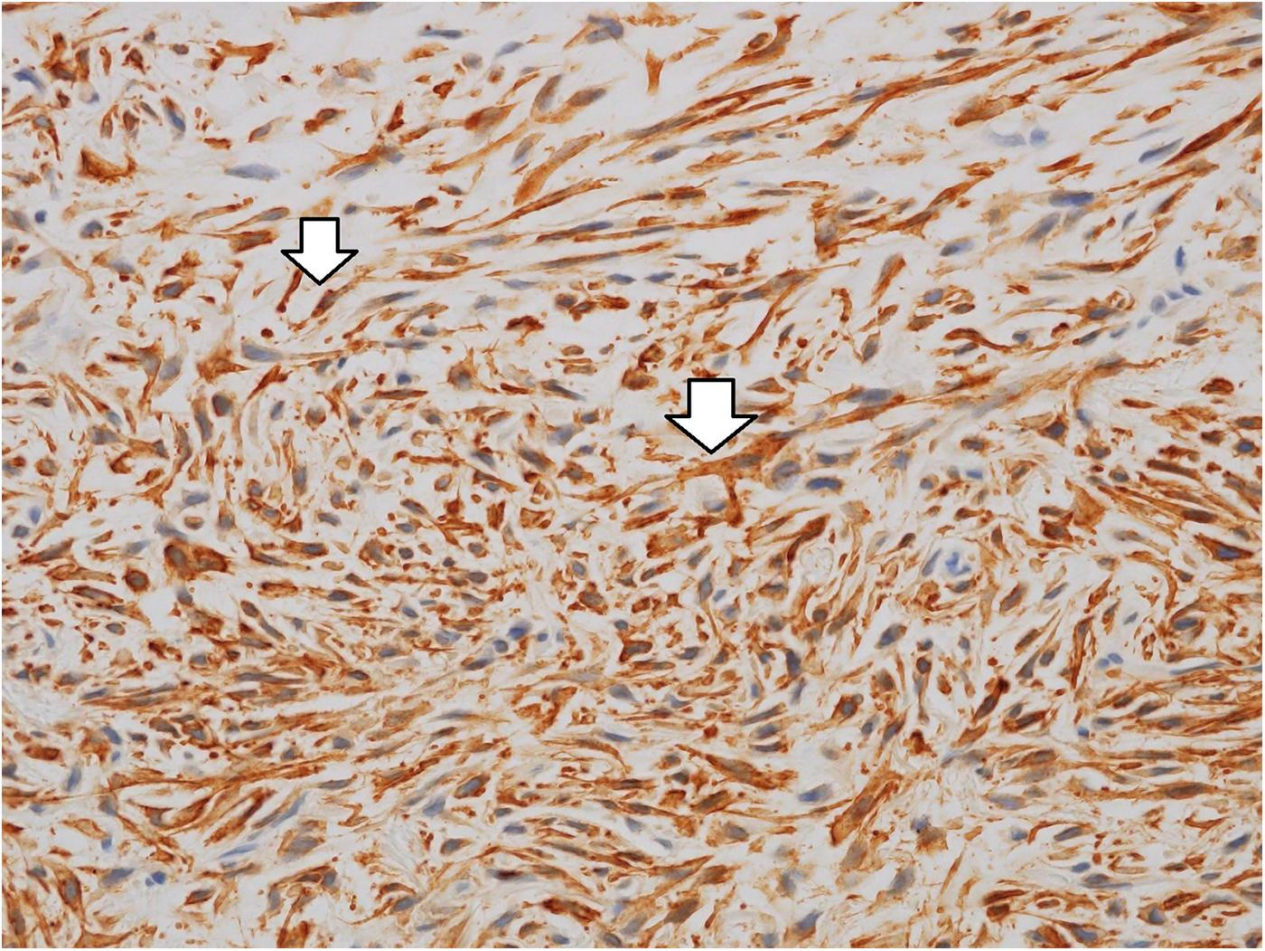
Metastatic low-grade myofibroblastic sarcoma in the lung. Immunohistochemical staining for smooth muscle actin shows diffuse and strong reactivity of the spindle cell cytoplasm (white arrow).

As the role of adjuvant chemotherapy or radiotherapy in this disease remains undetermined, close surveillance at 3–6-month intervals was planned through the hemato-oncology department. After 4 years and 1 month of no evidence of disease, tumor recurrance was detected on chest CT screening as a 1.5-cm right upper-lobe apical lung nodule with lymph node enlargement (Figure 2). She subsequently underwent right upper-lobe wedge resection via video-assisted thoracoscopic surgery. Permanent biopsyrevealed a tumor measuring 2.0 cm × 1.7 cm × 0.7 cm with clear resection margins, consistent with metastatic LGMFS. Immunohistochemistry was positive for smooth muscle actin (SMA) (Figure 3) and negative for CD31, CD34, and desmin. Figure 3 shows immunohistochemical staining of the metastatic lung lesion, in which the cytoplasm of the tumor cells exhibits brown coloration indicative of SMA positivity.

During the 3 years following lung surgery, the patient remained under regular surveillance at 6-month intervals, with no evidence of local recurrence or distant metastasis until she presented with palpable neck nodules. Breast evaluation revealed no recurrence. Ultrasonographyshowed nodules in the neck lymph node measuring 2.6 cm and 1.8 cm, and CNB confirmed metastatic myofibroblastic sarcoma (MFS). This recurrence occurred 6 years and 11 months after the wide excision of the breast. The patient was scheduled for consultation with the hemato-oncology department to initiate palliative chemotherapy; however, 10 days later, she presented to the emergency department with hemiplegia. Brain MRI revealed an infarction in the territory of the right middle cerebral artery bifurcation, which was assumed to be a malignant infarction. Multiple metastatic nodules were also observed in the skull and sclera. Brain surgery was not feasible, and the patient died 2 days later. The total follow-up period was approximately 7 years.

## Discussion

MFS was first reported by Wargotz et al. (7), and low-grade spindle cell sarcoma with myofibroblastic differentiation was analyzed by Mentzel et al. (3). According to histopathological grading, LGMFS is classified as an intermediate (rarely metastasizing) tumor (8). LGMFS is characterized by low-grade malignant potential, a propensity to recur locally, a predilection for the head and neck region, and a low likelihood of distant metastasis (3, 5, 9). Treatment primarily involves surgical excision with margins to prevent local recurrence (8). This is based on a small number of case reports, and an appropriate surgical excision margin for LGMS has not yet been clearly established (1). Comparative analyses of patients who underwent treatment with surgery and radiation versus those who did not demonstrated no significant differences in the overall or disease-free survival on either univariate or multivariate analyses (2). Previous reports suggest a limited role for chemotherapy in the management of LGMFS (10, 11).

LGMFS of the breast is rare. To date, 21 cases of mammary LGMFS have been reported. Of these, 10 patients underwent mastectomy and seven underwent local excision. Four patients did not receive definitive surgical treatment. As reported, seven of 21 myofibroblastic sarcomas originated from breast metastases to other organs. Among these, four developed lung metastases, two developed bone metastases, and one patient developed both lung and brain metastases. Almost all patients underwent either local excision or mastectomy; however, the use of postoperative therapy, chemotherapy, or radiotherapy varied. Only one of the 21 patients received both radiotherapy and chemotherapy, one received chemotherapy alone, and three received radiotherapy alone. The patient who underwent combined chemotherapy and radiotherapy showed no recurrence or metastasis. However, patients who received chemotherapy alone developed metastases limited to the lungs. Among the three patients who received radiotherapy alone, none experienced local recurrence, although one patient developed lung metastasis. Local recurrence was observed in two patients who underwent mastectomy and in three patients who underwent wide excision. Unlike conventional LGMFS, mammary LGMFS showed a higher frequency of distant organ metastasis (7 of 21) than local recurrence (5 of 21). In this case, surgery was performed with adequate margins, and lung metastasis developed 4 years after surgery without evidence of local recurrence. Three years after lung surgery, multiple neck lymph node metastases occurred, followed by death from tumor emboli, again without local recurrence. We followed-up the patient for approximately 7 years, which represents the longest follow-up duration to date for mammary LGMFS. Further studies with long-term follow-up are required to clarify the prognosis of this disease.

This case report is limited by its retrospective nature and the inability to generalize findings from a single case. Comprehensive conclusions regarding optimal treatment or surveillance strategies cannot be drawn. Furthermore, because mammary LGMFS is extremely rare, the existing literature is limited to small case series and isolated reports, making comparative interpretation challenging.

In conclusion, the diagnosis of MFS by CNB can be challenging because various spindle cell neoplasms exhibit smooth muscle or myofibroblastic differentiation. In our case, the phyllodes tumor was first diagnosed by CNB. Therefore, surgical resection is recommended to establish an accurate diagnosis. Due to the scarcity of information on this disease, there are no consensus guidelines for standard treatment. Although wide excision appears to be the most reasonable treatment strategy, the effects of chemotherapy or radiotherapy on the management of this disease remain uncertain. Mammary MFS seems to be more prone to metastasis to the lungs than to other organs. Further studies are required to clarify the pathophysiology and the possibility of metastasis to establish proper guidelines.

This study was approved by the Institutional Review Board of Pusan National University Yangsan Hospital (IRB No. 55-2025-010).

## Data Availability

The raw data supporting the conclusions of this article will be made available by the authors without undue reservation.
